# Characterization of microRNAs expression profiles in human dental-derived pluripotent stem cells

**DOI:** 10.1371/journal.pone.0177832

**Published:** 2017-05-18

**Authors:** Xiaobing Tan, Qingyuan Dai

**Affiliations:** 1 Department of Cariology and Endodontics, First People’s Hospital of Yunnan Province, Kunming, China; 2 Department of Cariology and Endodontics, Affiliated Hospital of Kunming University of Science and Technology, Kunming, China; 3 Department of Cardiology, First Affiliated Hospital of Kunming Medical University, Kunming, China; Università degli Studi della Campania "Luigi Vanvitelli", ITALY

## Abstract

Induced pluripotent stem cells (iPSCs) technology provides a powerful means to generate and regenerate unlimited pluripotent stem cells directly from body tissue cells. Stem cells from apical papilla (SCAP) and Dental pulp stem cells (DPSCs) are present in ‘cell-rich zones’ within the dental pulp region, which are capable of regenerating pulp and dentin tissues *in vivo*. In this study, we investigated the difference of miRNAs expression in SCAPs and DPSCs before and after the reprogramming. Using miRNA microarray, 134 and 265 differentially expressed miRNAs in DPSCs- and SCAP-iPSCs were up-regulated compared to these before reprogramming. 117 specific miRNAs with enhanced more than 2-fold were identified in both DPSCs- and SCAP-iPSCs. Among the co-regulated miRNAs, miR-19a-3p, miR-92b-3p and miR-130b-3p showed the maximum difference, which had involvement in the cell cycle, TGF beta signaling pathway and epithelial mesenchymal transition. Using qRT-PCR analysis, the expression of miR-19a-3p, miR-92b-3p and miR-130b-3p indicated substantial increases in DPSCs-iPSCs and SCAP-iPSCs. The findings suggest that miRNAs play a part in the difference between DPSCs-iPSCs and DPSCs, as well as between SCAP-iPSCs and SCAP. The variation of miRNA expression in reprogrammed dental-derived pluripotent stem cells revealed different characteristics induced by iPSC generation.

## Introduction

In the past ten years, the establishment of induced pluripotent stem cells (iPSCs) has been as one of the breakthroughs in the field of stem cell research [1, 2]. This breakthrough discovery provides a powerful means to generate and regenerate unlimited pluripotent stem cells directly from body tissue cells [3]. Like embryonic stem cells (ESCs), iPSCs share the majority properties with embryonic stem cells, such as morphology, differentiation, DNA methylation, gene expression, unlimited self-renewal ability and potential to generate any differentiated cell type (pluripotency) [4]. As previous studies reported, the self-renewal and pluripotency properties of iPSCs are not only regulated by an array of protein-coding genes but also a group of noncoding microRNA (miRNA) genes [5, 6]. With ongoing advances in miRNA biology, using nonviral vectors, such as RNA and microRNAs (miRNAs), to generate iPSCs has also been revealed [7]. miRNA is ~20–22 nucleotides in length and well known to control extensive cellular functions via affecting the abundance and translation efficiency of cognate mRNA. miRNAs are expressed in a variety of cells, such as embryonic stem cells, iPS cells and somatic cells. A number of co-expressed miRNAs clusters, such as miR-302-367, and miR-290, were disclosed in embryonic stem cells, iPS cells and somatic cells [8].

Stem cells from apical papilla (SCAP) are originated from the developing tissue at the apex of a tooth root termed apical papilla [9]. Dental pulp stem cells (DPSCs) are present in ‘cell-rich zones’ within the dental pulp region [10]. They are capable of regenerating pulp and dentin tissues *in vivo*. In our previous study, we have reprogrammed the SCAP and DPSCs into to iPS cells [11]. However, the difference of miRNAs expression before and after the reprogramming remain largely unexplored. In this study, chip analysis technology was used to screen the differential miRNAs expression in reprogramming process of human dental iPS cells, thereby providing a basis for further research on the mechanism of miRNA and its target genes in the reprogramming process.

## Materials and methods

### Isolation, culture and identification of human DPSCs and SCAP in vitro

We collected 2 mandibular third molars from the same patient (age<20) indicated for extraction at department of oral and maxillofacial, first people's Hospital of Yunnan Province. All patients provided written informed consent, and the study was approved by the Ethics Committee of the first people's Hospital of Yunnan Province.

**Fig 1:**
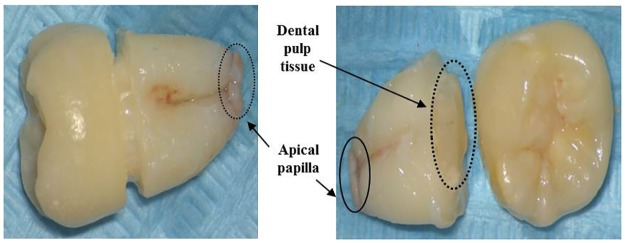
Dental pulp tissue and apical papilla isolated from third molars.

DPSCs and SCAP isolated from third molars (Fig 1) were culture in medium consisting of α-MEM (Gibco/Invitrogen, Grand Island, NY, USA), 10% fetal bovine serum (Thermo Scientific), 1% Glutamax (Gibco/Invitrogen, Grand Island, NY, USA) and 0.5% Penicillin/Streptomycin ((Invitrogen). At P3, DPSCs and SCAP were harvested by trypsinization with 0.05% trypsin (Invitrogen) upon reaching 90% confluence, and re-suspended in DPBS to reach a final cell density of 1.5× 10^6^ cells/ml. An amount of 200ul of cell suspension (1.5×10^6^ cells) was incubated in the dark for 30min at room temperature with fluoro isothycyanate-conjugated antibodies against CD24, CD34, CD45, stro-1 (all from Invitrogen), and phycoerythrin-conjugated antibodies against CD90, CD105, CD146, oct-4 (all from eBioscience) for specific surface antigens analysis by using flow cytometer. All analyses were standardized against negative control cells incubated with Isotype-specific IgG2ak-PE (Invitrogen). The results of flow cytometer were analyzed by using Summit, Version 5.1

### Transduction and reprogramming of DPSCs and SCAP

The transduction was carried out based on the instructions of the CytoTune-iPS Sendai Reprogramming kits. Two days before transfection, DPSCs and SCAP at P3 were seeded separately into wells of 6-well plates with 1 x 10^5^ cells each well. On the transfection day, appropriate volumes of each of the three CytoTune 2.0 Sendai tubes were dissolved in 70uL medium were used for transduction of DPSCs and SCAP and then 130 uL medium was added after one day. On the second day, the medium were replaced by medium included fresh SeV. On the third day, the transfected cells were transferred to the 6-well plates covered with matrix and cultured with reprogramming medium. New DPSCs iPSC and SCAP iPSC colonies emerged in about three weeks. The clone were picked using the "cross method" and transferred into the new plates and cultured with PSC-easy medium included 10μM Y27632 (Selleck, Shanghai, China). H9 (P53) routinely cultured was as a standard control.

### miRNA microarray analysis

90% confluent P3 human DPSCs /SCAP and P12 DPSCs/SCAP-iPSCs were collected for the miRNA isolation. Total RNA (including miRNA) was isolated using TRIzol reagent (Invitrogen, Carlsbad, CA, USA) and purified with RNeasy Mini Kit (Qiagen) following the manufacturer’s instructions. The purity and quantity of the isolated RNAs were assessed by 1% formaldehyde-agarose gel electrophoresis and spectrophotometry (NanoDrop ND-1000; Thermo Scientific, Wilmington, DE). The RNAs from P3 human DPSCs /SCAP and P12 DPSCs/SCAP-iPSCs have been respectively mixed and put into microarray analysis. After further processing, the isolated miRNAs were labeled with Hy3^™^ by using the miRCURY^™^ Hy3^™^/Hy5^™^ Power labeling kit (Exiqon, Vedbaek, Denmark). The Hy3^™^-labeled miRNAs were then hybridized to individual miRCURYTM LNA microRNA Arrays (v.18.0, Exiqon). Microarray images were obtained by an Axon Genepix 4000B microarray scanner (Axon Instruments, Union City, CA, USA), and processed and analyzed by the accompanying Axon Genepix Pro 6.0 software. Signal values of more than 30 in all samples were extracted for further analysis. After normalization, differentially expressed miRNAs were screened based on the fold change and P-value between cells before and after reprogramming. miRNAs with an adjusted p value less than 0.05 (ANOVA) were identified as differentially expressed miRNAs in these different reprogrammed cells.

### Quantitative validation of miRNA using qRT-PCR

Total RNA from DPSCs /SCAP before and after reprogramming were used as template to synthesize cDNA with the One Step PrimeScript miRNA cDNA Synthesis kit (TaKaRa). The qPCR assay was conducted with reagents from the SYBR Green Real-Time PCR kit (TaKaRa), and the qRT-PCR mixture contained SYBR Premix Ex Taq II, forward and reverse primers (Table 1), UnimiR qPCR primer, cDNA, ROX reference dye. The reactions were conducted by using ABIPrism 7000 Sequence Detection System (Applied Biosystems) with initial enzyme activation at 95°C for 30 s, followed by 45 cycles of denaturation at 95°C for 5 s and annealing and extension at 60°C for 34 s. The threshold cycle (Ct) was determined by using the default threshold settings. All experiments were performed in triplicate and repeated three times. The expression level of genes of interest was normalized against housekeeping gene U6. The fold change was calculated by using the equation 2^−ΔΔ^CT method.

**Table 1:** miRNA-specific primers used in the qRTPCR.

miRNA	Forward primer (5’-3’)
U6	F: 5’GCTTCGGCAGCACATATACTAAAAT3’ R: 5’CGCTTCACGAATTTGCGTGTCAT3’
miR-19a-3p	GSP: 5’GGGGGGGTGTGCAAATCT3' R: 5' GTGCGTGTCGTGGAGTCG3'
miR-92b-3p	GSP: 5’GGTATTGCACTCGTCCCG3’ R: 5'GTGCGTGTCGTGGAGTCG3’
miR-130b-3p	GSP: 5'GGGCAGTGCAATGATGAAA3' R: 5’GTGCGTGTCGTGGAGTCG3’

### Bioinformatic prediction and pathway analysis of miRNAs target

The differentially expressed miRNAs targets were determined by using the miRanda (http://mirdb.org/miRDB/), microCosm(http://www.ebi.ac.uk/enright-srv/microcosm/htdocs/targets/v5/) and targetscan (http://www.targetscan.org/) [12]. To determine the biological and functional properties of all the differentially expressed miRNAs target, they were mapped with Gene Ontology Terms (http://geneontology.org/). The target genes of these miRNAs were also mapped to KEGG pathways. The enrichment results are presented in a “bubble plot” using the ggplot2 package [13]. Additionally, the biological functions associated with these networks are also provided.

## Results

### Primary culture of human DPSCs and SCAP and flow cytometric analyses

**Fig 2:**
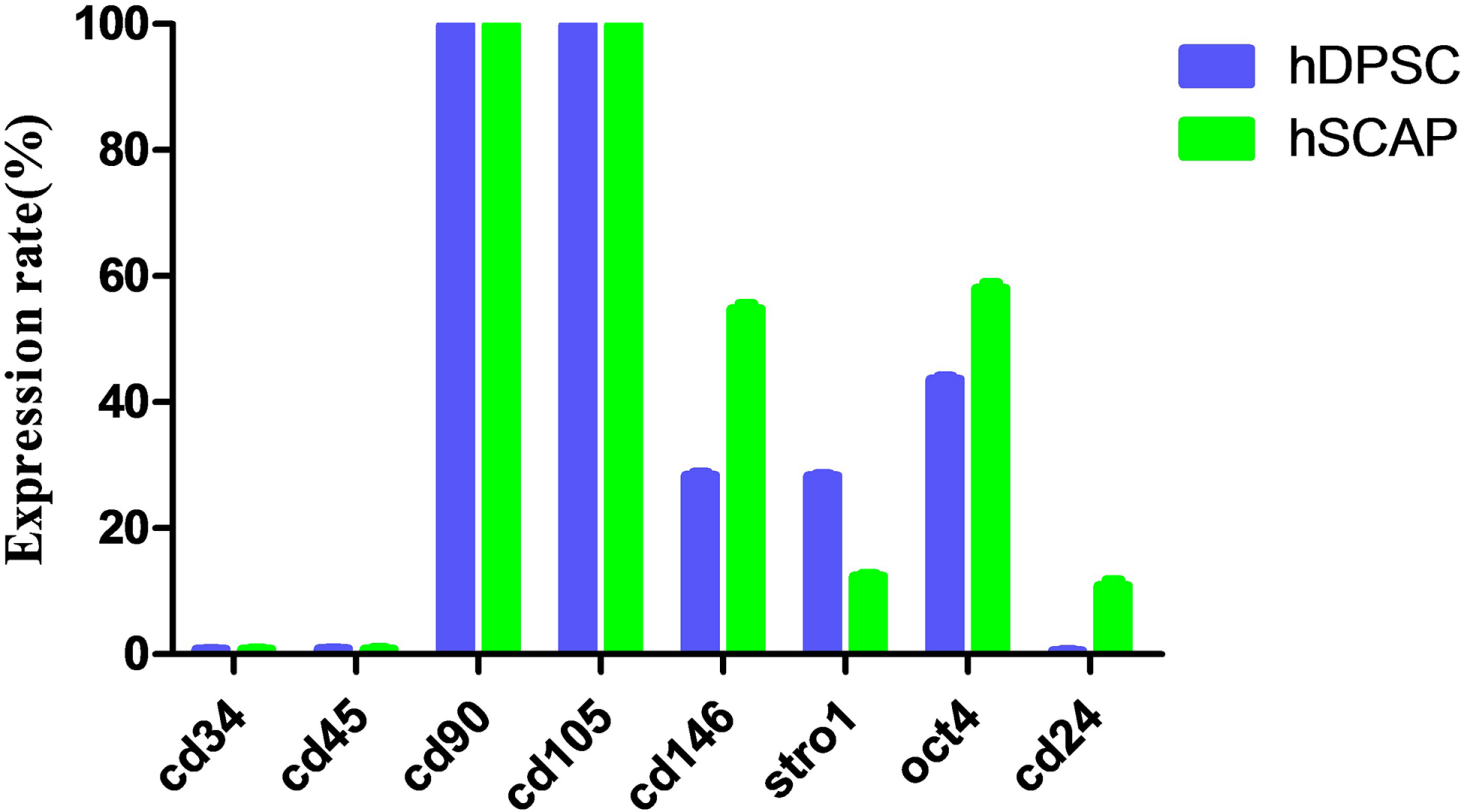
The expression rate of surface markers in DPSCs and SCAP.

The isolated human DPSCs and SCAP in vitro were cloned after culturing for four days. The flow cytometric analyses showed that DPSCs and SCAP were both negative for the haematopoietic surface markers of CD34 and CD45. On the contrary, DPSCs and SCAP were positive (about 100%) for CD90 and CD105. DPSCs (28.4±0.56) % and SCAP [(54.8±0.96) %] were also shown strong positive for CD146 (Fig 2). At the same time, DPSCs and SCAP were positive for STRO-1[(28.3±0.4) % and (12.4±0.46)%] and OCT-4 [(43.6±0.66)% and (58±1.22)%] respectively (Fig 2). CD24 was only expressed in the SCAP [(10.9±1.06)%] (Fig 2).

### Generation of DPSCs-iPSCs and SCAP-iPSCs

**Fig 3:**
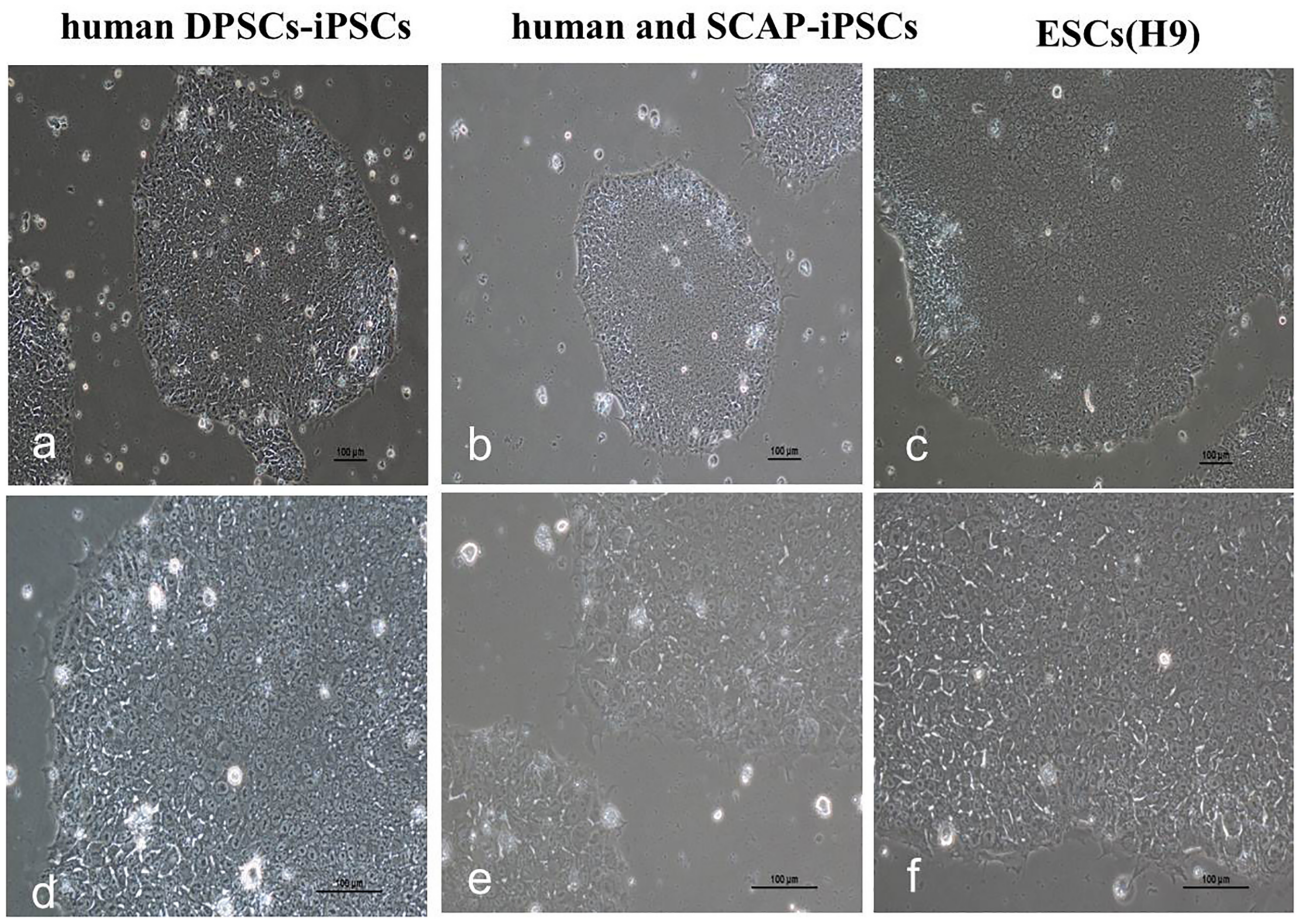
The culture of human DPSCs-iPSCs, SCAP-iPSCs and ESCs *in vitro*.

The transfected DPSCs and SCAP were seeded onto Matrigel. Within three to four weeks after transfection, ESC-like colonies emerged on Matrigel (Fig 3a–3e), which were primary iPS cells (P0). The iPS clones were transferred to the new matrix via cross method (P1) and passaged in four to five days. The culture of human DPSCs-iPSCs and SCAP-iPSCs in vitro were as indicated in Fig 3.

### Differential expression of miRNAs between DPSCs/ SCAP and DPSCs-iPSCs/ SCAP-iPSCs

**Fig 4:**
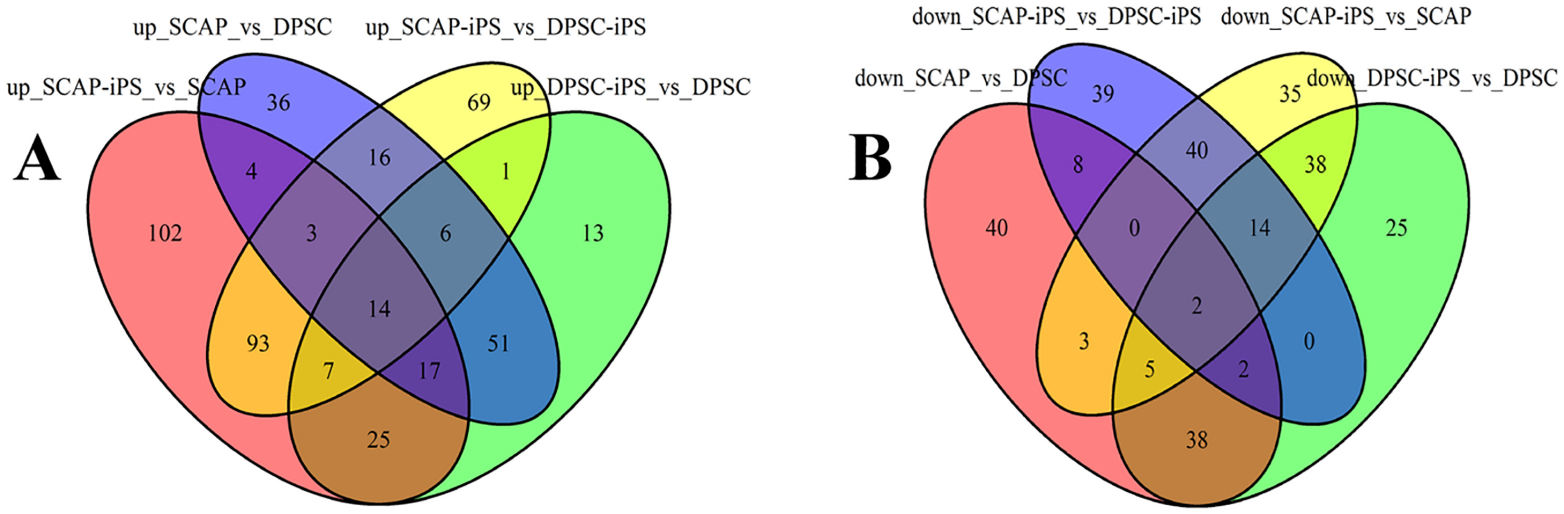
The Venn diagrams of the number of miRNA genes expressed in human DPSCs/SCAP-iPSCs. A: Up-regulated miRNA genes. B: Down-regulated miRNA genes.

**Fig 5:**
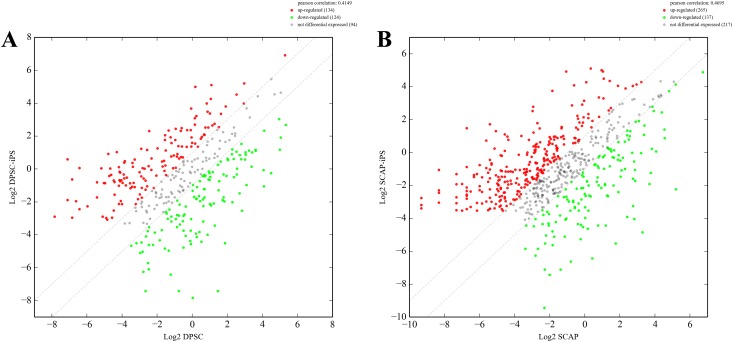
Comparison of the expression levels between DPSCs and DPSCs-iPSCs (A) as well as SCAP and SCAP-iPSCs (B). The differentially expressed miRNA genes were filtered using P≤0.05 and |log2 ratio|≥1 as a threshold. The red points represent upregulated genes, and the green points indicate downregulated genes. The gray spots represent no significant difference.

A total of 1201 miRNA genes were all determined by using miRanda, microcosm and targetscan (S1 Table). The venn diagrams of human DPSCs/SCAP-iPSCs showed the differentially expressed miRNA in DPSCs-iPSCs vs. DPSCs, SCAP-iPSCs vs. SCAP, DPSCs vs. SCAP and DPSCs-iPSCs vs. SCAP-iPSCs (Fig 4). Of the miRNA genes analyzed by miRNA microarray, 134 miRNA genes in DPSCs-iPSCs were up-regulated and 124 were down-regulated compared to those in DPSCs (Fig 5A). In SCAP-iPSCs, 265 miRNA genes showed upregulated expression and 137 downregulated expression compared to those in SCAP (Fig 5B). Among these up-regulated miRNA genes, 117 enhanced more than 2-fold were identified in both DPSCs-iPSCs and SCAP-iPSCs. Among these 117 up-regulated miRNA genes, miR-19a-3p, miR-92b-3p and miR-130b-3p showed the maximum difference between reprogrammed iPS cells and DPSCs/SCAP. These results implied that the difference between reprogrammed cells and DPSCs/SCAP might be attributed, at least in part, to differently expressed miRNAs. The following target prediction analysis on miR-19a-3p, miR-92b-3p and miR-130b-3p using bioinformatic neural nets are summarized in Table 2 (S1 Table).

**Table 2:** Putative target genes of the six differentially expressed miRNAs.

	Target gene	Gene description
hsa-miR-19a-3p	ACTN1	actinin, alpha 1
	ALPK2	alpha-kinase 2
	ATP10A	ATPase, class V, type 10A
	BCL3	B-cell CLL/lymphoma 3
	BTG1	B-cell translocation gene 1, anti-proliferative
	CAB39L	calcium binding protein 39-like
	CAST	castor zinc finger 1
	DHRS3	dehydrogenase/reductase (SDR family) member 3
	DLC1	deleted in liver cancer 1
	DOCK10	dedicator of cytokinesis 10
	ELK3	ELK3, ETS-domain protein (SRF accessory protein 2)
	FAM114A1	family with sequence similarity 114, member A1
	FAM69A	family with sequence similarity 69, member A
	FBXO8	F-box protein 8
	FLNC	filamin C, gamma
	FOSL1	FOS-like antigen 1
	FOXF2	forkhead box F2
	HIPK3	homeodomain interacting protein kinase 3
	LRIG3	leucine-rich repeats and immunoglobulin-like domains 3
	LRRTM2	leucine rich repeat transmembrane neuronal 2
	MAP2K3	mitogen-activated protein kinase kinase 3
	MAP3K12	mitogen-activated protein kinase kinase kinase 12
	MICAL2	microtubule associated monoxygenase, calponin and LIM domain containing 2
	NRP2	neuropilin 2
	PDE4A	phosphodiesterase 4A, cAMP-specific
	PIK3CA	phosphoinositide-3-kinase, catalytic, alpha polypeptide
	PNRC1	proline-rich nuclear receptor coactivator 1
	PON2	paraoxonase 2
	POSTN	periostin, osteoblast specific factor
	PRKAA1	protein kinase, AMP-activated, alpha 1 catalytic subunit
	REEP3	receptor accessory protein 3
	RHOB	Rho-related BTB domain containing 1
	SH3KBP1	SH3-domain kinase binding protein 1
	SMARCA2	SWI/SNF related, matrix associated, actin dependent regulator of chromatin, subfamily a, member 2
	SNX17	sorting nexin 17
	SPOCK1	sparc/osteonectin, cwcv and kazal-like domains proteoglycan (testican) 1
	SPRYD3	SPRY domain containing 3
	ST3GAL5	ST3 beta-galactoside alpha-2,3-sialyltransferase 5
	THBS1	thrombospondin 1
	TMEM45A	transmembrane protein 45A
	TNFRSF12A	tumor necrosis factor receptor superfamily, member 12A
	TNIP1	TNFAIP3 interacting protein 1
	TOR1B	torsin family 1, member B (torsin B)
	WNT1	wingless-type MMTV integration site family, member 1
	ZFPM2	zinc finger protein, multitype 2
	ZMYND11	zinc finger, MYND-type containing 11
hsa-miR-92b-3p	AATK	apoptosis-associated tyrosine kinase
	ACAN	aggrecan
	ADAMTSL1	ADAMTS-like 1
	ARHGEF17	Rho guanine nucleotide exchange factor (GEF) 17
	BAHCC1	BAH domain and coiled-coil containing 1
	DKK3	dickkopf homolog 3 (Xenopus laevis)
	GATA2	GATA binding protein 2
	GRIA3	glutamate receptor, ionotrophic, AMPA 3
	HIPK3	homeodomain interacting protein kinase 3
	ITGA5	integrin, alpha 5 (fibronectin receptor, alpha polypeptide)
	JPH2	junctophilin 2
	KIF5B	kinesin family member 5B
	KLF2	Kruppel-like factor 2 (lung)
	MYH9	myosin, heavy chain 9, non-muscle
	MYO1B	myosin IB
	NKX2-3	NK2 homeobox 3
	OAZ3	ornithine decarboxylase antizyme 3
	PAX9	paired box 9
	POLK	polymerase (DNA directed) kappa
	RAB23	RAB23, member RAS oncogene family
	RGS3	regulator of G-protein signaling 3
	RRBP1	ribosome binding protein 1 homolog 180kDa
	SLC39A6	solute carrier family 39 (zinc transporter), member 6
	SMAD6	SMAD family member 6
hsa-miR-130b-3p	ACVR1	activin A receptor, type IB
	APCDD1	adenomatosis polyposis coli down-regulated 1
	CLIP1	CAP-GLY domain containing linker protein 1
	COL6A3	collagen, type VI, alpha 3
	ELK3	ELK3, ETS-domain protein (SRF accessory protein 2)
	FRMD6	FERM domain containing 6
	INHBB	inhibin, beta B
	LYSMD2	LysM, putative peptidoglycan-binding, domain containing 2
	MAP3K12	mitogen-activated protein kinase kinase kinase 12
	MLLT6	myeloid/lymphoid or mixed-lineage leukemia (trithorax homolog, Drosophila); translocated to, 6
	NDEL1	nudE nuclear distribution gene E homolog (A. nidulans)-like 1
	NRP2	neuropilin 2
	PFKFB3	6-phosphofructo-2-kinase/fructose-2,6-biphosphatase 3
	PPARG	peroxisome proliferator-activated receptor gamma, coactivator 1 beta
	STARD13	StAR-related lipid transfer (START) domain containing 13
	STX12	syntaxin 12
	TMEM9B	TMEM9 domain family, member B
	WNT1	wingless-type MMTV integration site family, member 10A

### Analysis of up-regulated miRNAs expressed in DPSCs-iPSCs and SCAP-iPSCs

**Fig 6:**
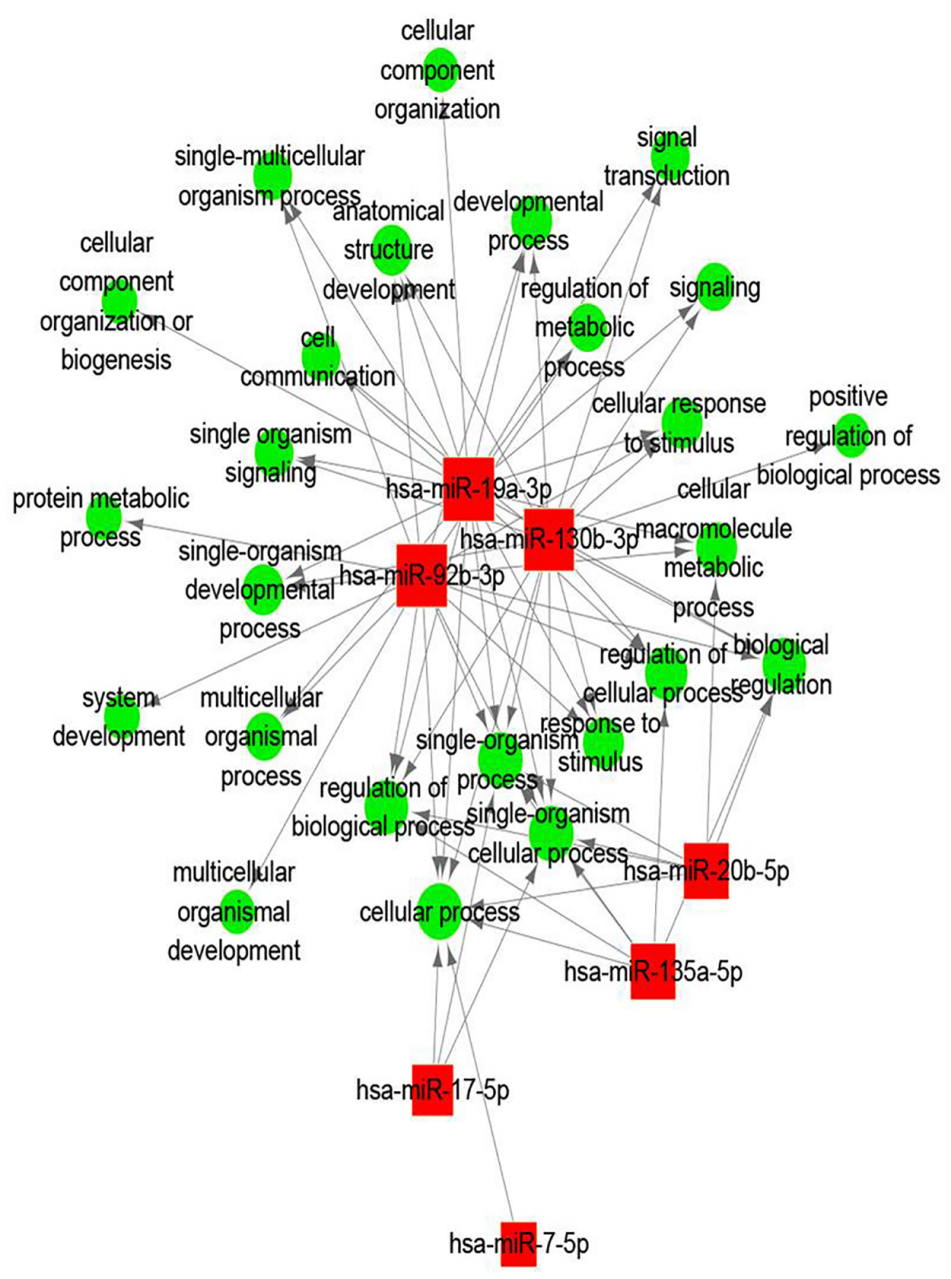
The significantly enriched Gene Ontology (GO) terms of the up-regulated expressed miRNAs. The red nodes represent the target genes of the differentially expressed miRNAs, and the green nodes represent the molecular function of the GO terms.

**Fig 7:**
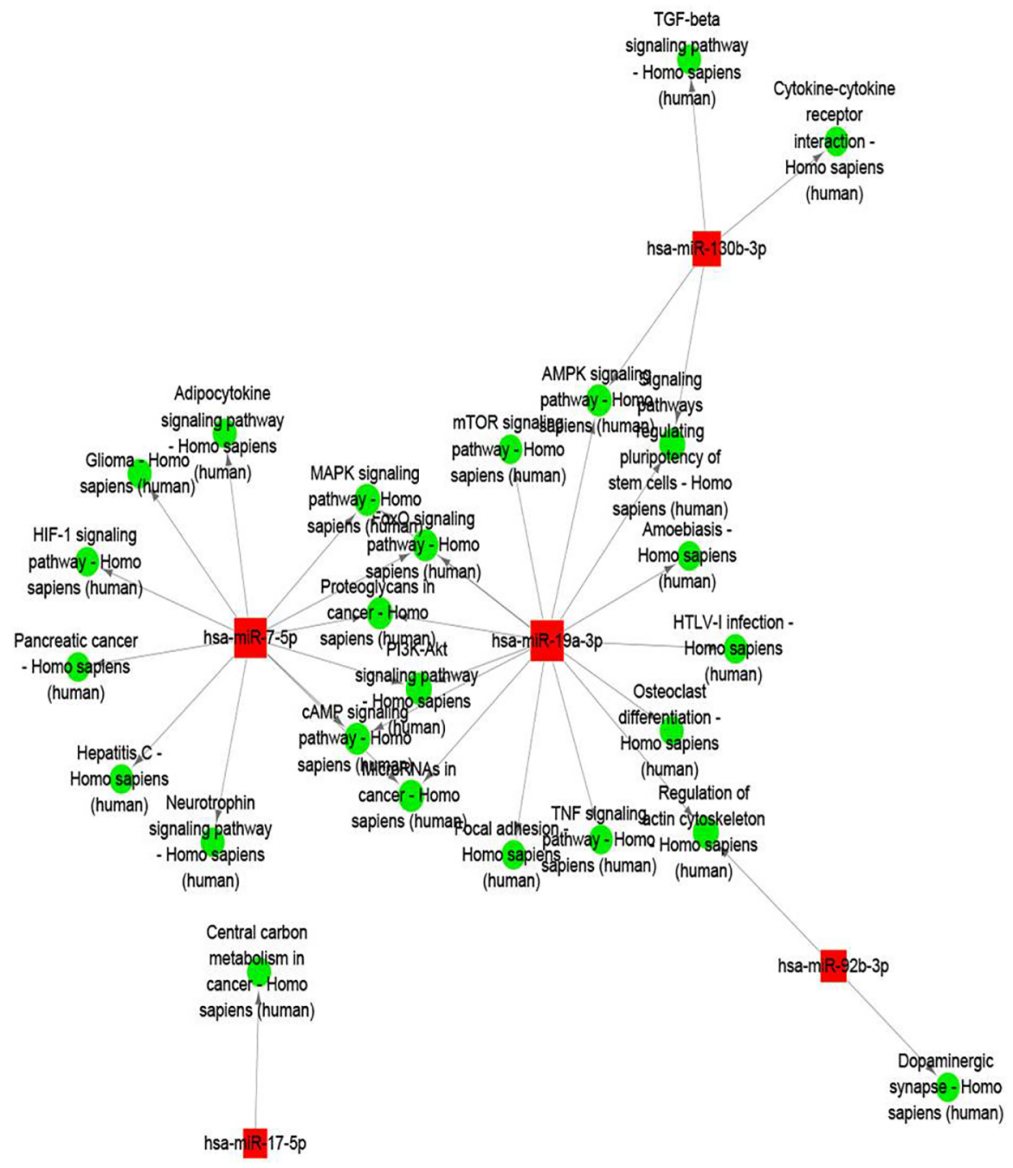
The predicted target genes involve the KEGG pathway-mediated regulatory network in the reprogramming process of human dental iPS cells. The red nodes represent the target genes of the differentially expressed miRNAs, and the green nodes represent the involved KEGG pathway.

Further bioinformatics analyses are conducive to investigate the roles of the three highly expressed miRNAs in the reprogramming process of human dental iPS cells. Gene Ontology and KEGG pathway enrichment analyses were used to predict the biological functions of the miRNA targets. GO analysis of the miRNA targets showed that miR-19a-3p, miR-92b-3p and miR-130b-3p were mostly involved in the function of cell biological process, metabolic regulation and stimulating reaction (Fig 6 and S2 Table). The biological functions generated from the top 3 up-regulated miRNA demonstrated their involvement in various pathways. The miR-19a-3p, it was related with 15 pathways, such as signaling pathways regulating pluripotency of stem cells, focal adhesion and amoebiasis. And the miR-92b-3p was involved in the regulation of actin cytoskeleton and dopaminergic synapse. As for the miR-130b-3p, it was showed the participate in the cytokine-cytokine receptor interaction, AMPK and TGF-beta signaling pathway and signaling pathways regulating pluripotency of stem cells (Fig 7 and S3 Table). Hence, our prediction that the differentially expressed miRNAs have the capability to target various components in these crucial pathways makes them promising molecular targets for the induction of iPSCs.

### Validation of the three differentially expressed miRNAs by using qRT-PCR

**Fig 8:**
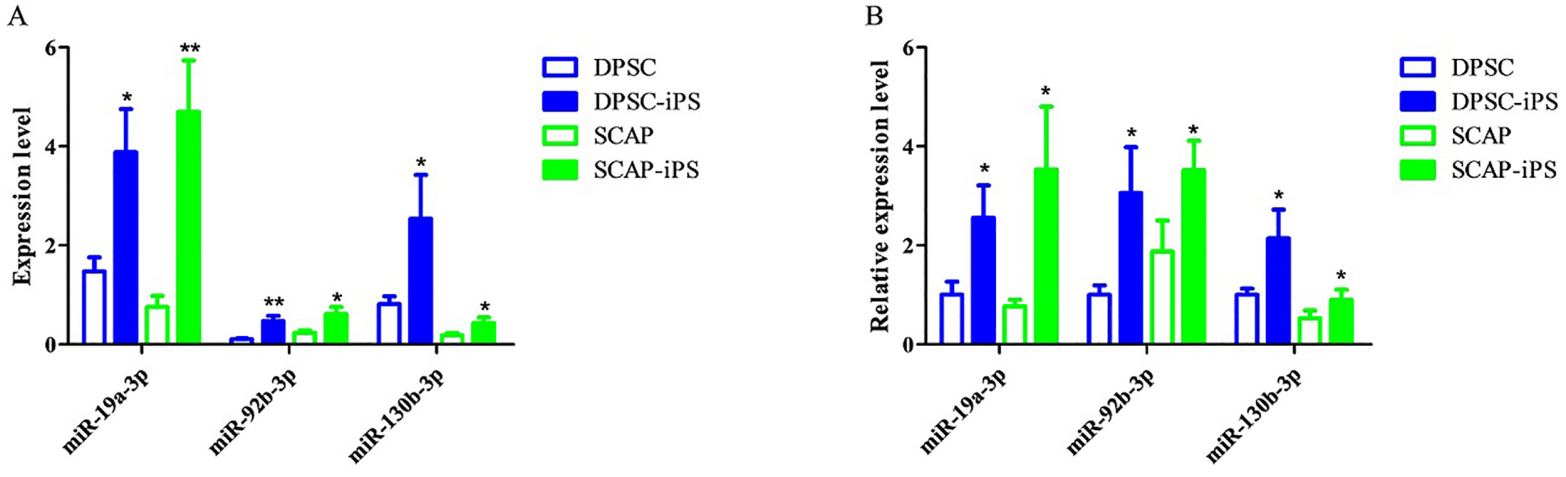
Comparison of the levels of three differentially expressed miRNAs detected on microarray (A) and by qRT-PCR(B). t-test was implemented for comparisons between dental-derived pluripotent stem cells before and after reprogrammed. *, p<0.05; **, p<0.01.

As shown in Fig 8A, the results detected on microarray indicated that the miR-19a-3p, miR-92b-3p and miR-130b-3p displayed substantial increase expression in DPSCs-iPSCs and SCAP-iPSCs. The distinct expressions of the three miRNAs found in DPSCs-iPSCs and SCAP-iPSCs were further confirmed by using qRT-PCR analysis. As a result, they all showed consistent tendency as those detected on microarray (Fig 8B).

## Discussion

Stem cells have the ability to rescue and/or repair injured tissue and could be isolated from the human body. Among these, DPSCs and SCAP are relatively easily obtainable and exhibit high plasticity and multipotential capabilities [14]. A previous had addressed that their isolation, selection, and differentiation are of great importance [15]. Using a Sendai virus vector, we successfully generated the DPSCs-iPSCs and SCAP-iPSCs with typical iPSCs characteristics, fibroblastoid morphology, proliferation, multipotent differentiation capability and the expression of a typical set of haematopoietic surface markers.

It has been clearly revealed that varied types of cell vary not only in the expression of their coding genes, but also in the expression of their non protein-coding portion of the genome [16]. Currently, one of the non protein-coding portion that has received increasing attention is noncoding RNAs (ncRNAs), which is of crucial functional relevance in several mechanisms of gene regulation, both for normal development and physiology, and for human diseases [17]. The most well characterized ncRNA are lncRNAs and miRNAs. As the best-known class of ncRNAs, miRNAs is able to regulate many target genes and control gene expression through translational repression and degradation [18]. In the present study, we compared differences in miRNAs expression between DPSCs/SCAP and DPSCs-iPSCs/SCAP-iPSCs to recognized pluripotent specific miRNAs. 134 up-regulated miRNA genes were identified in DPSCs-iPSCs and 265 in SCAP-iPSCs. Among these up-regulated miRNA genes, 117 miRNAs with more than 2-fold enhancement were found in both DPSCs-iPSCs and SCAP-iPSCs. These results were suggested that the role played by the differently expressed miRNAs in the difference between reprogrammed cells and DPSCs/SCAP. Based on these up-regulated miRNAs, 3 specific miRNAs, namely hsa-miR-19a-3p, hsa-miR-92b-3p and hsa-miR-130b-3p, with the maximum difference between reprogrammed cells and DPSCs/SCAP were entered into further discussion.

The hsa-miR-19a-3p, was reported as a miRNA involved in the leukotriene biosynthesis via targeting 5-Lipoxygenase in a cell type- and stimulus-specific manner [19]. This gene is said to function as vital feedback in signal molecules between irradiated GBM stem-like cells and non-irradiated cells [20]. Interestingly, there are also reports revealing that miR-19a and miR-19b, which are oncogenic in human malignancies, are the key components in human fibroblastic cell reprogramming and enhanced human fibroblast reprogramming, in the presence or absence of cMyc [21]. In this study, miR-19a-3p was highly expressed in two dental stem cells (DPSCs-iPSCs and SCAP-iPSCs), suggesting that hsa-miR-19a-3p may have potent to stimulate induction of iPSCs. Furthermore, the target gene analysis showed that this miRNA was targeted forty genes (Table 2). Among these targeted genes, WNT was relevant to governing transcription of pluripotent genes, self-renewal and differentiation in most of the SCs found in adult tissues [22]. Dissimilar levels of WNT signaling were resulted in distinct lineage-specific differentiation properties in human ESCs [23]. Our further prediction revealed that hsa-miR-19a-3p was involved in signaling pathways regulating pluripotency of stem cells, focal adhesion and amoebiasis. Further study is warrant as for the relationship between hsa-miR-19a-3p and these signaling pathway.

Hsa-miR-92b-3p has been reported to target several important lipogenic gene to regulate the networks of lipid deposition [24]. There are also report indicating that miR-92b-3p is to be specifically associated with neural progenitors [25]. The computational data in our study predicted that hsa-miR-92b-3p is connected to many targeted genes, such as AATK, GATA2, HIPK3, MYO1B, OAZ3 and SMAD6. Further prediction showed that hsa-miR-92b-3p was involved in the regulation of actin cytoskeleton and dopaminergic synapse. This miRNA showed elevated expression in reprogrammed dental cells, implying that miR-92b-3p may be capable of stimulating induction of iPSCs.

hsa-miR-130b-3p has been known to promotes CD133^+^ liver tumor-initiating cell growth and self-renewal via tumor protein 53-induced nuclear protein 1 [26]. It was also reported that this miRNA was targeted the insulin-like growth factor (IGF-1) in the epithelium [27]. TGF-b signaling reportedly functions in both hES and mES cell self-renewal, and FGF2, a widely used growth factor for ES cell culture, induces TGF-b ligand expression and suppresses BMP-like activities [28]. Our analysis on the hsa-miR-130b-3p showed its involvement in the cytokine-cytokine receptor interaction, AMPK and TGF-b signaling pathway and signaling pathways regulating pluripotency of stem cells, suggesting that hsa-miR-130b-3p may be a potential inducer of iPSCs.

## Conclusions

In conclusion, we report differentially expressed miRNAs between reprogrammed cells and DPSCs/SCAP. The specific expression of miRNAs, namely hsa-miR-19a-3p, hsa-miR-92b-3p and hsa-miR-130b-3p, which were prediction to be related to the cell cycle, TGF beta signaling pathway and epithelial mesenchymal transition, may reflect the difference between naturally pluripotent cells and reprogrammed cells. The abilities and mechanism of these specific expression of miRNAs in the reprogramming process of human dental iPS cells warrant further exploration.

## Supporting information

S1 Table The differentially expressed miRNAs targets predicted by the miRanda microCosm and targetscan and the up-regulated and down-regulated miRNA genes. (XLSX)

S2 Table Gene Ontology analysis of miRNA targets. (XLS)

S3 Table Pathway analysis for miRNA genes that up-regulated miRNA. (XLS)
